# Clinical Application of Human Urinary Extracellular Vesicles in Kidney and Urologic Diseases

**DOI:** 10.3390/ijms17071043

**Published:** 2016-06-30

**Authors:** Giuseppe De Palma, Fabio Sallustio, Francesco Paolo Schena

**Affiliations:** 1 C.A.R.S.O. Consortium, University of Bari, Valenzano 70010, Italy; g.depalma@nephro.uniba.it; 2 Schena Foundation—European Research Center for Kidney Diseases, Valenzano 70010, Italy; 3 Department of Emergency and Organ Transplantation, University of Bari, DETO, Bari 70124, Italy; fabio.sallustio@uniba.it

**Keywords:** extracellular vesicles, urine, kidney diseases

## Abstract

Extracellular vesicles (EVs) have been isolated in different body fluids, including urine. The cargo of urinary EVs is composed of nucleic acids and proteins reflecting the physiological and possibly pathophysiological state of cells lining the nephron and the urinary tract. Urinary EVs have been confirmed to contain low amounts of various types of RNA that play a role in intercellular communication by transferring genetic information. This communication through EV RNAs includes both continuation of normal physiological processes and conditioning in disease mechanisms. Although proteins included in urinary EVs represent only 3% of the whole-urine proteome, urinary EVs can influence cells in the renal epithelia not only by delivering RNA cargo, but also by delivering a wide range of proteins. Since urine is a readily available biofluid, the discovery of EVs has opened a new field of biomarker research. The potential use of urinary EV RNAs and proteins as diagnostic biomarkers for various kidney and urologic diseases is currently being explored. Here, we review recent studies that deal in identifying biomarker candidates for human kidney and urologic diseases using urinary EVs and might help to understand the pathophysiology.

## 1. Introduction

The discovery of extracellular vesicles (EVs) in human plasma dates back to 1967, when this subcellular fraction was identified by electron microscopy by Wolf and was shown to consist of small vesicles, with a diameter between 20 and 50 nm, originating from platelets and termed “platelet dust”. This “platelet dust” was capable to induce thrombin formation similarly to platelets [1].

Sixteen years later, two independent groups observed that the transferrin receptors associated with 50 nm vesicles were jettisoned from maturing blood reticulocytes into the extracellular space [2,3].

In 1996, over a decade after the detection of exosomes in reticulocytes, other investigators [4] established that major histocompatibility complex (MHC)-II-enriched multivesicular endosomes (termed “MIIC”) in B lymphocytes fused with the plasma membrane to release exosomes bearing MHC-II, and these exosomes were able to present the peptide–MHC-II complex that activated the T cell response. Then, it was shown that dendritic cells produced exosomes [5] with the capacity to stimulate T cell response [6].

Later, EVs were purified from almost all mammalian cell types, including stem cells [7,8,9,10,11,12,13], primary cells of the immune and nervous systems [14,15,16,17,18] as well as numerous cancer cell lines [19,20,21]. Notable, the secretion of EVs is not limited to mammalian cells but it has also been recognized in lower eukaryotes and prokaryotes [22,23]. Of course, EVs were in the first time regarded as membrane debris with no real biological meaning. Then, the importance of EVs in intercellular communication—via the transfer of proteins, lipids and nucleic acids—was confirmed in numerous studies [24,25,26].

The extracellular vesicles (EVs) are classified on the base of their cellular origin and/or biological function or on their biogenesis (Table 1). The three main classes of EVs are exosomes, microvesicles and apoptotic bodies. In this review we are focusing on the first two classes of EVs. Both are cell-derived vesicles that are delimited by a lipid bilayer, ranging from 30 to 1000 nm in diameter depending on their origin. While exosomes are believed to derive from the endolysosomal process, the microvesicles are thought to be generated by budding from the cellular membrane [27].

The protein content of different types of EVs typically reflects that of the parent cells and is enriched in certain molecules, such as adhesion molecules, membrane trafficking molecules, cytoskeleton molecules, heat shock proteins, cytoplasmic enzymes, signal transduction proteins, cytokines, chemokines, proteinases and cell-specific antigens. Furthermore, EVs contain mRNAs, non-coding RNAs (ncRNAs), including microRNAs (miRNAs) and retrotransposon RNA transcripts, and even single-stranded DNA (ssDNA), double-stranded DNA (dsDNA), mitochondrial DNA, and oncogene amplifications (i.e., cmyc) [14,32,33].

Numerous mechanisms have recently been identified to control exosome biogenesis, thus facilitating protein and RNA cargo sorting to produce exosomes with a precise biochemical composition [24,34,35,36]. Despite these recent advances on EVs, the terms “exosome” and “microvesicle” have been used interchangeably in many available studies.

Some investigators have studied EVs with a size less than 100 nm in diameter defining them exosomes, while others considered only EVs with a cut off value of 150–200 nm in diameter. This has become a main issue in many reports. Some papers use the term “microvesicles” instead of “exosomes” [30], others use the terms “exosome-like vesicles” [37], or “prostasomes” [38,39] (in reality some are a subset of the exosome family). Databases such as ExoCarta and Vesiclepedia have emerged to address this issue [40], though it remains largely unresolved as the data entered in these databases relies on the terminology used by authors in their published papers. There are discrepancies in reports in relation to the size of EVs.

Exosomes and microvesicles are not one type of vesicles secreted by cells. Some agree that the term microvesicle covers a broader range of vesicles with a diameter from 100 nm up to 1 μm [41]. On the other hand, unlike the multivesicular body origin of exosomes, microvesicles originate from the plasma membrane of eukaryotic cells after stimulation or apoptosis. Microvesicle biogenesis involves phospholipid distribution and dynamic contraction of the plasma membrane by cytoskeletal actin/myosin, regulated by ADP-ribosylation factor 6 [30]. It may be that there are populations of membrane-shed microvesicles with a diameter less than 100 nm.

Practically all cell types liberate EVs as well as tumour cells [42], antigen presenting cells [4,43], T cells [44], stem cells [11] and epithelial cells [45]. Therefore, they are present in the plasma and other body fluids, including breast milk, semen, saliva, urine and sputum. They have a key role not only in the regulation of normal physiological processes, such as stem cell maintenance [8], tissue repair [46], immune surveillance [4] and blood coagulation [47], but also in the pathology of several diseases.

## 2. Urinary Extracellular Vesicles

EVs were first found in human urine [48] and afterwards, were shown usually secreted from all nephron segments [49]. Consistent with their origin, renal tubular epithelial cells have multivesicular bodies at the apical surface, and urine exosomes include apical membrane proteins from every cell type along the nephron [50,51]. There is interaction between exosome-like vesicles and primary cilia of renal epithelial cells [50], and there is support for in vitro uptake of exosomes by the renal cortical collecting duct cell line [51], leading to theory that exosomes may provide intrarenal proximal-to-distal transapical renal tubular epithelial signalling through RNA transfer.

Urinary EVs can be obtained by a two-step differential ultracentrifugation process. This method allows efficient isolation of EVs with a high degree of purity, though it is laborious and time-consuming. However, density-gradient ultracentrifugation also cleans samples from other contaminants such as soluble proteins [52], thus contributing to a better definition of the specific composition of EVs. Later, precipitating agents (including PEG and commercial reagents) have been used to enrich EVs from different biological fluids, including urine [53]. However, these reagents precipitate most of the contaminants found in the ultracentrifugation pellet. Importantly, these procedures co-isolate contaminants that may be incorrectly identified as EV-related proteins or RNAs [54]. This is particularly relevant in urine, where major common components of the fluid such as the polymeric Tamm–Horsfall protein and other proteins (mainly albumin) are present in proteinuric samples. Therefore, specific pre-isolation techniques are recommended to reduce or eliminate the presence of Tamm–Horsfall protein before any EV isolation/determination in urine samples [55]. Size-exclusion chromatography has been widely used to separate complex mixtures of molecules of different size. It has been used after ultracentrifugation to isolate EVs from urine samples [56] or following a conventional centrifugation to eliminate cell debris and apoptotic bodies. Urine samples can be concentrated using ultrafiltration and loaded on a size-exclusion chromatography column [57].

Considering the limitations in the urinary EV isolation and the amount of variability between samples, strong normalisation methods are needed to allow valid studies. Human urinary EV proteins are normalized to urine creatinine that is the traditional variable, although creatinine excretion rate varies widely among humans. Alternatively, the excretion of specific exosomal proteins can be normalized to the excretion of proteins specifically associated with exosomes and often absent in EVs other than exosomes such as PDCD6IP or TSG101. They are part of the endosomal sorting complex required for transport (ESCRT) machinery that has been recently shown to play a direct role in exosome biogenesis [58]. This assumes that the normalising protein urinary excretion does not change in different disease states and there is no biological/clinical relevance to a change in the number of EVs within the sample. There is no consensus regarding the optimal approach for normalization and studies are needed to evaluate alternatives among normalizing factors.

Typical populations of EVs are being studied using: (i) immunoblotting that shows the presence of EV proteins; (ii) transmission electron microscopy; and (iii) flow cytometry that provides information on the relative presence of surface proteins. It is important to note that the variability in number of EVs excreted in urine within and across people is not clearly defined and high volumes of urine are required in order to generate useful quantities of EVs. At present, counting a precise number of EVs in a given sample is a challenge. However, technological advances are promising for the future such as the Nanoparticle Tracking Analysis (NTA) that can rapidly size and directly count EVs in urine and identify surface marker proteins [59]. A study demonstrated that when whole urine samples were applied to the Nanosight chamber, NTA can determine the size and concentration distribution of nanoparticles sized between 26 nm and 700 nm and a significant percentage of the overall particle distribution is within the expected exosomal size range (20–100 nm). Labelling with two different exosomal proteins it has been established the presence of exosomal sized particles in unprocessed urine. It provides “proof of concept” data demonstrating that NTA can rapidly quantify exosome concentration without time-consuming sample processing [60]. Alternatively, tunable resistive pulse sensing (tRPS) allows for reliable and rapid measurement of EVs in small sample volumes. It is based on the detection of nanosized particles upon their movement through a nanosized pore [61]. A study has directly compared NTA, tRPS and flow cytometry by analysing the size distributions of urinary EV [62]. Interestingly, comparable EV quantifications by NTA and tRPS were reported, whereas the flow cytometry-based EV quantification was 15 times lower. Moreover, new and more precise methods are being studied, such as the electric field-induced release and measurement. It can at the same time both disrupt exosomes, releasing their content, and monitor on-site the harboured exosomal RNA/proteins biomarkers [63].

## 3. Clinical Applications of Urinary Extracellular Vesicle RNAs and Proteins

Human urinary EVs contain mRNA that, like their protein cargo, derive from all regions of the nephron [64]. Thus, urinary exosomal shuttle RNA (esRNA) could be a tool to examine kidney cellular transcriptional changes in health and diseases. In an interesting study on the specificity of exosomal RNA as a biomarker, the urinary exosomes of the V-ATPase B1 subunit were found in knockout mice to faithfully reflect the loss of this specific subunit mRNA while normal amounts of the other V-ATPase B2 subunit and aquaporin 2 were detected [64,65].

It is important to note that the human urinary microvesicle nucleic acid load is dominated by ribosomal RNA reliable with a typical eukaryotic cell RNA profile together with the presence of prominent 18S and 28S rRNA peaks [64]. This is dissimilar to the profile reported for exosomes isolated from cultured cell lines that contained little or no 18S and 28S rRNA. Nevertheless, this difference could be due to the fact that they are contaminated with cellular debris. This is in contrast with that found by other investigators who have not detected 18S and 28S rRNA in urinary exosomes [66,67].

miRNAs are short (20–25 nucleotides) ncRNA molecules that inhibit a set of specific target mRNAs and regulate specific cellular proteins. They are a new class of disease biomarkers because certain miRNA species are organ specific. They are released from cells following injury [68] and are stable in a range of biofluids including urine [69]. Exosomes released from cell lines contain miRNAs, although exosomal miRNAs are distinct from cellular miRNAs in terms of relative abundance [70].

In addition, urinary EVs contain several kind of proteins secreted by the kidney tissue, some of which have sparked interest as potential diagnostic new markers of kidney disease in urinary EVs [71,72,73].

Urinary mRNA profiling has been proposed as a tool for biomarkers [74]. However, they are degraded by urinary RNase. Instead, the EV nucleic acids are in a notable stable form because EVs protect them from endogenous RNase activity.

For clinical applications, EV diagnostics are required to be: (1) fast; (2) simple and highly sensitive; and (3) able to detect specific endogenous biomolecules contained in the EVs.

Numerous studies in kidney and urologic diseases have described the presence of urinary biomarkers from EVs. Table 2 shows a summary of the current state of biomarkers.

### 3.1. Acute Kidney Injury

Proteomic analysis of urinary EVs has been used in a study investigating the changes in urine protein content associated with acute kidney injury [71]. The analysis, carried out by gel electrophoresis in two different rat models of renal injury, showed that protein levels changed earlier than the increase in serum creatinine. Fetuin A has been recognized in this report as a biomarker of acute kidney injury. These findings were validated by Western blotting using the samples from nine patients, but validation of these findings in a sufficiently large cohort is still pending [71]. Using two mouse models of acute kidney injury, cisplatin and ischaemia/reperfusion injury, Zhou et al. demonstrated a significant increase of the levels of activating transcription factor 3 (ATF3) in urinary EVs but not in whole urine after the induction of damage. Of note, the urinary vesicle marker ATF3 not only remained elevated for 24–48 h, but also increased before the raising of the serum creatinine, supporting the clinical interest for this biomarker. These results were subsequently demonstrated in patients with acute kidney injury, in whom the increase of ATF3 preceded the increase of the serum creatinine [75]. An opposite trend was observed for aquaporin-1, as its content in the urinary EVs rapidly declined both in a rat model of *ischaemia/reperfusion injury* and in patients immediately after kidney transplantation [73].

In the last years, several promising early biomarkers of acute kidney injury have been identified in urine, including neutrophil gelatinase-associated lipocalin (NGAL), interleukin-18 (IL-18) and kidney injury molecule-1 (KIM-1), that are in advanced stages of validation while despite the increasing number of studies on the EVs, urinary EV biomarker discovery remains in its infancy for insufficient number of patients involved in above mentioned studies.

### 3.2. Glomerular Diseases

Experiments have shown an increase in the levels of miR-26a in exosomes from patients of *lupus nephritis* and a positive correlation with urinary protein levels, suggesting its convenience as a predictive biomarker of lupus nephritis [76].

Urinary exosomes have recently been utilized for biomarker discovery in *IgA nephropathy*. Many urinary exosome proteins were identified including cytoplasmic, membrane and vesicle components. Among different expressed proteins, 4 were selected (aminopeptidase N, vasorin precursor, α1 antitrypsin and ceruloplasmin) as candidate biomarkers potentially useful in the differential diagnosis of IgA nephropathy from congenital thin basement membrane nephropathy [77].

Wilms Tumor 1 (WT1) is constitutively expressed on podocytes of healthy adult kidneys, and its expression decreases in kidney biopsies of patients with *primary focal segmental glomerulosclerosis* (FSGS) [78]. It has been found that urinary exosomal WT1 not only distinguishes between active FSGS and active steroid-sensitive nephrotic syndrome (SNSS) but also between active SNSS patients and SNSS patients in remission. In a small longitudinal study, urinary exosomal WT1 significantly decreased in SSNS after steroid treatment compared with pretreatment [72]. Unfortunately, this was not confirmed in paediatric patients with nephrotic syndrome in which WT1 levels in urinary EVs did not vary according to the responsiveness to the steroid therapy [96]. Other authors who isolated exosomal proteins from urine samples of patients with FSGS identify urinary podocalyxin, as a protein marker of patients with FSGS and nephrotic-range proteinuria. Podocalyxin decreased significantly in male FSGS patients compared with healthy normal, age-matched men [79].

A study showed that urinary podocytes from patients with *glomerular kidney disease* such as IgA nephropathy and lupus nephritis showed elevated levels of ADAM10. Higher concentrations of this protein were detected in urine as well as in urinary exosomes [80].

A study on microRNA expression in urinary exosomes from type 1 diabetic patients with and without incipient *diabetic nephropathy* has demonstrated that in patients with microalbuminuria miR-130a and miR-145 were enriched, while miR-155 and miR-424 were reduced. This finding is of potential pathophysiological relevance because miR-145 is a glomerular marker of mesangial cells and is induced by TGF-β1 in this cell type [67]. Dipeptidyl peptidase 4 is localized on the surface of many cell types, including the endothelial cells, kidney epithelial cells, and T cells, where it has a binding partner and transmits intracellular signals. In fact, the kidney shows the highest levels of dipeptidyl peptidase 4 per organ weight. Thus, some researchers reported an increased excretion of dipeptidyl peptidase 4 in microvesicles from type 2 diabetic patients [81].

The protein composition of urinary EVs is significantly different between patients and controls. Among the total amount of 254 different proteins analysed, 25 were significantly altered in diabetic nephropathy and validation studies confirmed that a panel of three of those proteins (AMBP, MLL3 and VDAC1) could be markers of the disease [82].

Until today markers in glomerulonephritis are miRNA [97] or chemokine/proteins; urinary EVs would allow at the same time to analyse RNA and protein for generating combined biomarkers that would increase diagnostic ability.

### 3.3. Tubular Diseases

In a study using a patient with *autosomal recessive polycystic kidney disease*, analysis of the urinary exosome-like vesicles identified 552 proteins, of which polycystin-1 and 2, cystin-1 and ADP-ribosylation factor like protein 6 were implicated in cystic diseases [50].

A report described the multiplex quantitative proteomic analysis of urinary EVs from patients with *autosomal dominant polycystic kidney disease* (ADPKD) [83]. The authors identified 83 differentially expressed proteins, many of which were involved in cytoskeleton regulation, Ca^2+^-activated signaling, cell division, cell differentiation, and Wnt signalling pathways. In particular, the ezrin/radixin/moesin family and CDC42 were significantly upregulated in ADPKD, reflecting the altered cytoskeleton regulation of tubular epithelial cells in ADPKD patients. Moreover, this study showed that expression of AQP2 reduced in ADPKD, consistent with the impaired urinary concentrating ability in these patients.

Despite their power in understanding functional aspects of EVs, investigation of urinary EV proteins show some limitations because they constitute only a subproteome.

### 3.4. Chronic Kidney Disease

In an exploratory study, osteoprotegerin was found in tubular cell-secreted exosome-like particles and urinary exosomes. This soluble protein was augmented in urinary exosome-like vesicles from patients with *chronic kidney disease*, including those with *diabetic nephropathy*, *IgA nephropathy* and *ADPKD* [84]. This finding was associated with upregulation of osteoprotegerin mRNA in kidney tissue [98]. Recently, Lv et al. have found decreased miR-29 family levels in exosomes isolated from urine of patients with chronic kidney disease, including those with biopsy proven diabetic nephropathy. It correlated positively with eGFR and negatively with degree of *tubulointerstitial fibrosis* [85]. Moreover, miR-29c was shown to potentially serve as predictor of early *fibrosis* in *lupus nephritis* [86]. Lv et al. also showed that the exosomal mRNA level of CD2AP was downregulated in kidney disease patients compared with healthy controls and correlated with renal function, level of proteinuria and severity of *renal fibrosis* [87].

These studies suggest that it is possible to find a match between cultured cells-derived EV content and body fluid-derived EV content.

### 3.5. Renal Transplantation

A recent study assessed the urinary exosomal mRNA expression of NGAL, IL-18, KIM-1 and cystatin C in the urine of patients with *kidney transplantation*. No correlation between exosomal mRNA levels and the seven-day creatinine reduction ratio (CRR7) was found, while the urinary proteins NGAL and IL-18 reflected the CRR7 [89]. It has been recently shown that levels of urinary CD133+ EVs are reduced in patients with end-stage kidney disease, possibly indicating that these vesicles are only released by functioning renal tissue. Indeed, in *transplanted patients*, CD133+ EVs were present at low levels in the first day after transplantation, to increase thereafter [88].

These studies demonstrate that it is possible to select the type of EVs secreted according to the cell of origin, even if packaging of mRNA in EVs is selective, and is not necessarily representative of mRNA in the parent cells responsible for biomarker production.

### 3.6. Cancer

Several companies are developing dedicated commercial assays for the detection of EVs in plasma or serum samples and in urine of patients with neoplasia (i.e., EVs in urine of patients with prostate cancer express mRNA encoding PCA3 and the TMPRSS2:ERG gene fusion products which are up-regulated in cancer cells) [90].

Other studies have also investigated on the proteomic cargo of prostate-derived extracellular vesicles from urine. Increased levels of integrin alpha-3 and integrin beta-1 have been found in urine exosomes of patients with metastatic prostate cancer, compared with patients with nonmetastatic disease or those with benign prostate hyperplasia [91]. Likewise, catenin delta associated with extracellular vesicles has been found at high levels in urine [92].

Few studies have addressed the potential use of urinary EVs as a diagnostic tool for renal cell carcinoma (RCC). A comparative analysis of urinary EVs performed using a hyphenated microLC-Q-TOF-MS (capillary liquid chromatography, quadrupole, time-of-flight mass spectrometry) platform demonstrated a differential lipid composition between the exosomes of patients with RCC and those of healthy control subjects [93]. Proteomic analysis of urinary EVs has facilitated the identification of an RCC-specific “fingerprint”, containing proteins such as matrix metalloproteinase-9, podocalyxin, dickkopf-related protein 4, carbonic anhydrase IX and ceruloplasmin [94]. EVs are able to transfer genetic information between cells, as they carry functional RNA transcripts, implying that the EV transcriptome might contain markers for the cell of origin. Our group found that urinary EV mRNA pattern was significantly different in RCC patients compared to healthy subjects and identified some EV mRNAs potentially suitable as gene signature. These mRNAs were decreased in urinary EVs of patients [99]. These preliminary studies suggest that urinary EVs could provide a tool for identifying new biomarkers of RCC.

Bladder cancer is another important neoplasia of the urinary tract. A study investigated the potential application of the RNA content of urinary EVs in the diagnosis of bladder cancer. Using microarray technology followed by PCR validation, Perez at al. generated a list of differentially expressed genes in urinary EVs from patients with cancer compared with those from cancer-free controls. LASS2 and GALNT1, which encode proteins involved in cancer progression and metastasis, were found only in EVs from the urine of patients with bladder cancer [95].

These studies show that currently the use of EV markers for tumour diagnosis can only be an initial useful clue but validation studies in large cohorts of patients are needed to determine their clinical use.

## 4. Conclusions

In this review we have illustrated some current aspects of the urinary EVs. They are released from all nephron segments, and contain various biological molecules ranging from RNA to proteins.

The detection of EVs is challenging because of their small and heterogeneous size range, high concentration, and heterogeneity in composition and morphology. At present, there is not one single method which can accurately phenotype, size, and enumerate the whole range of EVs. In addition, a more rigorous characterization of EVs using a combination of methods is likely needed (cytometry, electron microscopy, and full RNA and protein profiling). This will help to better explore the biology of EVs and their relationship to disease.

Urinary EVs have been confirmed to contain low amounts of RNAs [100] whose quantification is particularly challenging [101]. They are typically present at low concentrations and have a wide range of lengths (≈15 to thousands of nucleotides), with a prominent population of small RNAs (<200 nts) which play an essential part in intercellular communication by transferring genetic information. This communication through EV RNAs includes both continuation of normal physiological processes and conditioning in disease mechanisms. To date, many studies demonstrate the role of mRNAs and miRNAs from urinary EVs but researchers still continue to improve the ability to obtain larger quantities of these nucleic acids from urinary EVs.

Although proteins included in urinary EVs represent only 3% of the whole-urine proteome [102], these studies indicate that urinary EVs can influence cells in the renal epithelia not only by delivering RNA cargo, but also by delivering a wide range of proteins that may control/contribute to the gene expression. In fact, the proteome of urinary EVs is being studied for searching candidate markers of kidney diseases.

In conclusion, these studies show the possibility of distinguishing kidney and urologic disease patients from healthy people by the identified EV biomarker candidates. Urinary EVs are a suitable material for diagnosis because they are easy to collect and represent the pathophysiological state of kidneys. A rapid urinary assay of predictive EV biomarkers could provide to the clinician a support for the diagnosis and may allow a faster choice for adequate therapy. They may replace in the future the kidney biopsy. Finally, their use may have important scientific and commercial perspectives.

## Figures and Tables

**Table 1:** Characteristics of the extracellular vesicles.

Vesicle Type	Origin	Size (nm)	Markers ^1^	Contents	References
Exosomes	Endolysosomal pathway; intra-luminal budding of multivesicular bodies and fusion of multivesicular body with cell membrane	30–100	Tetraspanins (such as CD9 and CD63), ESCRT components (PDCD6IP and TSG101), flotillin	mRNA, miRNA and other non-coding RNAs; cytoplasmic and membrane proteins including receptors and major histocompatibility complex (MHC) molecules	[28,29]
Microvesicles	Cell surface; outward budding of cell membrane	100–1000	CD40 ligand, ARF6, VAMP3	mRNA, miRNA, non-coding RNAs, cytoplasmic proteins and membrane proteins, including receptors	[27,30]
Apoptotic bodies	Cell surface; outward blebbing of apoptotic cell membrane	50–5000	Extensive amounts of phosphatidylserine	Nuclear fractions, cell organelles	[31]

^1^ ESCRT: endosomal sorting complex required for transport; PDCD6IP: programmed cell death 6-interacting protein; TSG101: tumor susceptibility gene 101 protein; ARF6: ADP-ribosylation factor 6; VAMP3: vesicle-associated membrane protein 3.

**Table 2:** Suggested or studied urinary EV biomarkers in different renal and urological diseases.

Pathology	Biomarker ^1^	References
Acute kidney injury	Fetuin A, ATF3	[71,75]
Ischemia/reperfusion injury	ATF3, AQP1	[73,75]
Lupus nephritis	miR-26a	[76]
IgA nephropathy	aminopeptidase N, vasorin precursor, α1 antitrypsin, ceruloplasmin	[77]
Focal segmentary glomerulosclerosis	Wilms tumor 1, podocalyxin	[78,79]
Glomerular kidney disease	ADAM10	[80]
Diabetic nephropathy	miR-130, miR-145, miR-155, miR-424, dipeptidyl peptidase 4, AMBP, MLL3, VDAC1	[67,81,82]
Polycystic kidney disease	PKD1, PKD2, CYS1, ARF6, CDC42, AQP2	[50,83]
Chronic kidney disease	Osteoprotegerin	[84]
Renal fibrosis	miR-29c, CD2AP	[85,86,87]
Renal transplantation	NGAL, IL-18 and CD133	[88,89]
Prostate cancer	PCA3, TMPRSS2–ERG, integrin alpha-3, integrin beta-1, catenin delta	[90,91,92]
Renal cell carcinoma	Lysophosphatidylethanolamine metabolite, matrix metalloproteinase-9, podocalyxin, dickkopf-related protein 4, carbonic anhydrase IX and ceruloplasmin	[93,94]
Bladder cancer	LASS2 and GALNT1	[95]

^1^ ATF3: activating transcription factor 3; AQP1: aquaporin-1; ADAM10: Disintegrin and metalloproteinase domain-containing protein 10; AMBP: protein fragment of alpha-1-microglobulin/bikunin precursor; MLL3: isoform 1 of histone-lysine N-methyltransferase, VDAC1: voltage-dependent anion-selective channel protein 1; PKD1: polycystin-1; PKD2: polycystin-2, CYS1: cystin-1; ARF6: DP-ribosylation factor like protein 6; AQP2: aquaporin-2; CD2AP: CD2-associated protein; NGAL: neutrophil gelatinase-associated lipocalin; IL-18: interleukin-18; PCA3: prostate cancer associated 3; TMPRSS2-ERG: gene fusion product; LASS2: ceramide synthase 2; GALNT1: polypeptide *N*-acetylgalactosaminyltransferase 1.
